# Repurposing Amphotericin B and Its Liposomal Formulation for the Treatment of Human Mpox

**DOI:** 10.3390/ijms24108896

**Published:** 2023-05-17

**Authors:** Daniela Peruzzu, Katia Fecchi, Giulietta Venturi, Maria Cristina Gagliardi

**Affiliations:** 1 Center for Gender-Specific Medicine, Istituto Superiore di Sanità, Viale Regina Elena 299, 00161 Rome, Italy; 2 Department of Infectious Diseases, Istituto Superiore di Sanità, Viale Regina Elena 299, 00161 Rome, Italy

**Keywords:** lipid rafts, Mpox (monkeypox), drug repurposing, Amphotericin B

## Abstract

Mpox (monkeypox) is a zoonotic viral disease caused by the mpox virus (MPXV). Recently in 2022, a multi-country Mpox outbreak has determined great concern as the disease rapidly spreads. The majority of cases are being noticed in European regions and are unrelated to endemic travel or known contact with infected individuals. In this outbreak, close sexual contact appears to be important for MPXV transmission, and an increasing prevalence in people with multiple sexual partners and in men who have sex with men has been observed. Although *Vaccinia virus* (VACV)-based vaccines have been shown to induce a cross-reactive and protective immune response against MPXV, limited data support their efficacy against the 2022 Mpox outbreak. Furthermore, there are no specific antiviral drugs for Mpox. Host-cell lipid rafts are small, highly dynamic plasma-membrane microdomains enriched in cholesterol, glycosphingolipids and phospholipids that have emerged as crucial surface-entry platforms for several viruses. We previously demonstrated that the antifungal drug Amphotericin B (AmphB) inhibits fungal, bacterial and viral infection of host cells through its capacity to sequester host-cell cholesterol and disrupt lipid raft architecture. In this context, we discuss the hypothesis that AmphB could inhibit MPXV infection of host cells through disruption of lipid rafts and eventually through redistribution of receptors/co-receptors mediating virus entry, thus representing an alternative or additional therapeutic tool for human Mpox.

## 1. Introduction

Mpox (formerly named monkeypox) is a zoonotic viral disease caused by the mpox virus (MPXV).

MPXV is a double-stranded DNA virus of the genus Orthopoxvirus that belongs to the *Poxviridae* family and the *Chordopoxvirinae* subfamily [1]. There are two distinct phylogenetic subgroups, namely clade I (formerly called the Central African (Congo Basin) clade) and clade II (formerly called the West African clade). Additionally, clade II consists of two subclades, namely clade IIa and clade IIb. Different clades are associated with different geographical incidence locations and clinical features of the disease. Clade I exhibits greater virulence and causes a more severe disease with an average fatality rate of 10.6% compared to clade II, which has a case fatality rate of 3.6% [2]. Following the detection of the first human Mpox case in 1970 in the Democratic Republic of the Congo (DRC), infection cases were reported from West and Central African countries which have increased during the last decade, and large outbreaks have been identified mainly in the DRC [3,4]. Outside of Africa, an Mpox outbreak first occurred in the United States in 2003 [5,6], with 81 human cases in several states, all due to close contact with pet prairie dogs housed with MPXV-infected rodents imported from Ghana and no evidence of human-to-human transmission. Until 2022, imported human cases, all with a travel history from Nigeria, have been reported in the United Kingdom [7,8], Israel [9] and Singapore. In 2021, a family cluster occurred in the United Kingdom, with the primary case exposed in Nigeria [10,11].

Although many animal species were found to be infected with MPXV in nature, the specific animal reservoir of MPXV remains unknown. Some evidence suggests that native African rodents, such as Gambian giant rats (*Cricetomys gambianus*) and squirrels, are the major natural reservoir of the virus [12,13], which can sporadically transmit the virus to humans through spillover events. Humans are infected through direct contact or by eating an infected animal. Transmission between humans occurs, by large, because of respiratory droplets in prolonged face-to-face contact, bodily fluids and close skin-to-skin contact from active lesions [14,15].

Since May 2022, for the first time, a large multi-country outbreak of Mpox has been ongoing. Multiple cases of Mpox were identified in several non-endemic countries worldwide, not linked to traveling in endemic areas, nor with contact with imported mammals.

Specifically, in this outbreak, close sexual contact appears to be important for MPXV transmission, and an increasing prevalence in people with multiple sexual partners and in men who have sex with men has been observed [16,17]. At present, all sequences in the ongoing 2022–2023 Mpox outbreak are associated with clade IIb [18].

Mpox is a self-limiting disease with a duration of approximately 2–4 weeks and an incubation period of 6–13 days. Common symptoms in the prodromal phase include fever, headaches, fainting and rash development that are similar in cases of smallpox or measles. The rash phase begins on the face and extremities within 1 to 3 days of fever appearance, and then oral mucous membranes, genitalia, conjunctivae, cornea and lungs also become involved. Cutaneous lesions develop into pustular lesions which remain for 7–10 days before crust formation and desquamation after 1–2 weeks. Vesicular–pustular lesions are the principal clinical characteristics of Mpox disease, but lymphadenopathy is also a prominent feature that can be useful for diagnosing the disease [19]. To control the outbreak, public health measures must include strict isolation and supportive care of case patients for the duration of the infectious period, that is until the skin lesions dry up, become crusts and fall off. Where available, the contacts’ vaccination may be associated with this strategy, but it should not be considered as a replacement. Persons who may be at risk include personnel working in health facilities or laboratories involved in basic research or in diagnostic testing for Orthopoxvirus infections. Persons living in the same household or otherwise in close contact with a case, including but not limited to persons in the social and sexual networks of men who have sex with men, are also at risk in the current outbreak.

To date, vaccines initially developed against smallpox and based on VACV represent one of the possible measures to prevent and control the 2022 outbreak-causing Mpox. The World Health Organization (WHO) and the US Centers for Disease Control and Prevention (CDC) currently recommend the safer third-generation vaccine, a nonreplicating live variant of the VACV in high risk groups. Although VACV-based vaccines have been shown to induce a cross-reactive and protective immune response against MPXV, limited data support their efficacy against the 2022 outbreak-causing Mpox [20,21]. The epidemiological data indicate that most of the infected cases are those who did not receive smallpox vaccination in childhood, those had never been infected with Poxviruses or those who were born after the smallpox pandemic and eradication period.

Furthermore, there are no specific antiviral drugs for Mpox. Vaccinia immune globulin (VIG), a sterile solution of the purified gamma globulin G (IgG) fraction isolated from the plasma of vaccines, has been licensed by the United States Food and Drug Administration (FDA) in severe cases of human Mpox [22]. Anti-smallpox virus drugs (Tecovirimat, Cidofovir and Brincidofovir) approved by both the FDA and the European Medicines Agency (EMA) have shown efficacy against other Orthopoxviruses in animal models, including MPXV [23]. Tecovirimat is the only antiviral drug with an indication for the treatment of Orthopoxvirus infections, including Mpox, authorized by the EMA [24]. These drugs are considered good candidates for repurposing studies against Mpox, considering the conserved biology of Poxviruses [25].

New antiviral drugs for Mpox are urgently needed and one field of research to explore could be the use of compounds that target the entry step of the virus. Host-cell lipid rafts are small, highly dynamic plasma-membrane microdomains enriched in cholesterol, glycosphingolipids and phospholipids. Morphological lipid rafts can be classified into two distinct subtypes based on their protein composition: “planar lipid rafts” and “caveolae”. Caveolae are a flask-shaped membrane invagination with a concave configuration, and their key component is caveolin [26].

Planar lipid rafts have a typical flat and orderly structure and a smaller volume compared to the caveolae and the protein constituents that define their structure and function are flotillin proteins [27].

Cholesterol is the key dynamic glue that maintains lipid raft integrity by enhancing the order of other lipids and by regulating membrane fluidity and permeability. Moreover, the presence of cholesterol facilitates the lateral mobilization and the clustering of proteins and microdomains. In particular, physical segregation and aggregation of proteins in lipid rafts regulate their accessibility to effector molecules, such as cellular receptors, which are localized and concentrated by their downstream signaling pathways ligands [28]. In fact, lipid rafts form a sort of signal transduction platform by recruiting several signaling molecules, including glycosylphosphatidylinositol (GPI)-anchored proteins, protein kinases of Src family, transmembrane subunits of G-proteins, palmitoylated proteins and cholesterol-binding proteins [29].

Lipid rafts can rapidly assemble/disassemble and change their composition in response to intra- and extracellular stimuli [30] due to their dynamic and heterogeneous architecture. They coordinate many biological processes, including endocytosis, signal transduction and cell communication, phagocytosis and secretion, as well as pathogen adhesion/interaction [31].

Microbes select target cells and the site of binding depending on the surface receptors specific for cell types that allow their attachment, their internalization and their invasion of host cells. In these processes, microbes have evolved a number of different mechanisms and pathways based on host-cell type and environment. Although traditional clathrin-coated pit endocytosis is known to be the choice of numerous microbes to enter cells, host-cell lipid rafts have been recognized as crucial surface regions used predominantly or optionally by several bacterial, protozoan and viral pathogens as entry and signaling platforms [32,33]. These specialized membrane regions are involved in several steps of viral entry and in the late processes of viral protein transport, viral assembly, budding and release of virions [34]. They act as a platform to aggregate viral receptors or proteins and address the virus to the appropriate intracellular compartments. A large number of viruses have been shown to interact with lipid rafts to enter inside the cell or to seek refuge from the immune system and find a replication niche [35]. Their role has been ascertained in the life cycle of several viruses, including both enveloped Human Immunodeficiency virus (HIV)-1, Influenza virus, Flavivirus, Coronavirus, Poxvirus and non-enveloped Simian Virus 40 and Rotavirus, as well as DNA and RNA viruses, as confirmed by the antiviral effects of raft-disrupting agents on infection and viral replication [36]. Specifically, several compounds target membrane cholesterol, the dynamic glue that maintains raft integrity, and its removal makes rafts non-functional by causing the dissociation of most proteins. These drugs can also act by stimulating physiological pathways implicated in the efflux of cholesterol and by decreasing the supply of cholesterol through the inhibition of its biosynthesis [37]. In line with these studies, we previously demonstrated that the antifungal drug Amphotericin B (AmphB) inhibits *Candida albicans* phagocytosis by human monocytes, *Mycobacterium tuberculosis* infection of alveolar epithelial cells and Zika virus infection of Vero cells [38,39,40] through its capacity to sequester host-cell cholesterol and disrupt lipid raft architecture.

In this context, we discuss the hypothesis that a lipid-raft-disrupting agent, such as AmphB, could inhibit MPXV infection of host cells through the disruption of lipid rafts and eventually through redistribution of receptors/co-receptors mediating virus entry, thus representing an alternative or additional therapeutic tool for human Mpox.

## 2. Lipid Rafts and VACV Infection

**Figure 1:**
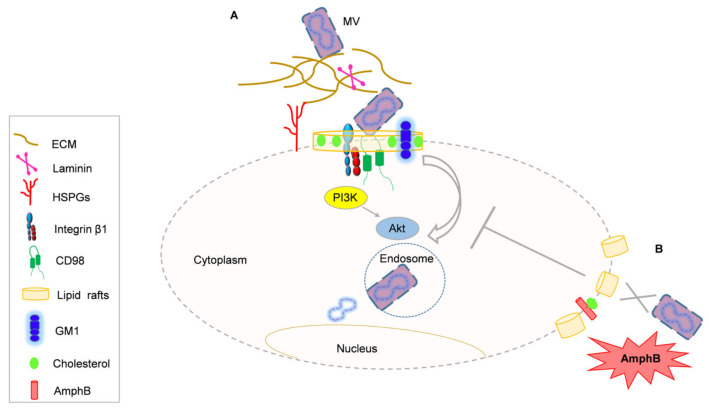
VACV/MPXV entry into host cells is inhibited by AmphB through lipid raft disruption. (**A**) After the attachment of the mature single-membrane virion (MV) to the extracellular matrix protein laminin and heparan sulfates proteoglycans (HPSGs), VACV exploits lipid rafts to infect host cells. VACV interacts with CD98, integrin b1 and ganglioside GM1, which are clustered into the raft platform to trigger PI3/Akt signaling. (**B**) AmphB disrupts lipid raft architecture by sequestering host-cell cholesterol. We speculate that AmphB through this mechanism would inhibit VACV/MPXV infection and PI3/Akt signaling.

VACV is the prototype of the Orthopoxvirus genus of the *Poxviridae* family and is considered a model system to study Poxvirus entry into host cells. It utilizes two different entry mechanisms into host cells, namely virus/host-cell membrane fusion and endocytosis. VACV produces a mature single-membrane virion (MV) and a double-membrane extracellular enveloped virion (EV) [41]. The entry mechanism of Vaccina MV is dependent on viral strains [42] and on host-cell types [43]. MV attachment to the host cell involves four proteins (A27, D8, H3, and A26) which bind to surface glycosaminoglycans (GAGs) and the extracellular matrix protein laminin. Specifically, H3 and A27 bind to heparan sulfates, A26 binds to laminin and D8 interacts with chondroitin sulfates (Figure 1). These interactions are important for the recruitment of MV virions, but they are not indispensable, because virions lacking these proteins preserve infectivity [44,45]. MV exploits a highly conserved entry fusion protein complex (EFC) for entry into host cells via membrane fusion. The EFC consists of eleven viral proteins which are highly conserved in all Poxviruses [46]. The underlying molecular mechanism responsible for VACV entry remains uncertain, likely due to the fact that Poxviruses are between the most complex virions. After attaching to the host cells, MV can also interact with specific cellular surface receptors that mediate virus internalization and host-cell entry. The receptors and co-receptors on the host-cell plasma membrane of VACV have been not definitively identified.

The involvement of lipid rafts in VACV entry has been already demonstrated. It has been shown that the cholesterol-depleting drug methyl-β-cyclodextrin (MBCD) inhibits VACV uncoating without affecting virion attachment, indicating that cholesterol-containing lipid rafts are crucial for viral entry into host cells. Several viral envelope proteins (A14, A17L, and D8L) are present in the cell membrane lipid raft fractions and colocalize with the ganglioside GM1, a lipid raft component on the cell membrane [47]. None of these viral proteins contain homologous motifs involved in lipid rafts’ association, suggesting the hypothesis that the aggregation of protein complexes through protein–protein interactions could account for VACV–lipid rafts association. Cell surface GAGs which have been shown to bind to VACV proteins are not involved in this interaction, although they can migrate into membrane rafts upon ligand binding [47]. Moreover, the lipid-raft-associated type II transmembrane glycoprotein CD98 has been shown to be required for VACV entry into HeLa cells and mouse embryonic fibroblasts. The knockdown of CD98 expression reduced MV infectivity without impairing VACV MV cell attachment, suggesting a role for CD98 in the post-binding step of virus entry [48]. CD98 is widely expressed on the cell surface and regulates several functions, including integrin signaling, which is involved in the host-cell entry of several viruses as Herpesviruses, Rotaviruses and Echovirus-1 [49]. The lipid-raft-associated protein integrin β1 has been shown to associate with VACV in the raft platform of HeLa cells and the knockdown of integrin β1 decreases VACV MV entry. VACV infection triggers the activation of PI3K/Akt signaling in an integrin β1-dependent manner, which is a crucial signaling pathway for virus endocytosis [50] (Figure 1). Several studies have shown that many viruses have evolved mechanisms to control this signaling to allow virus survival and transformation [51]. PIK3/Akt signaling pathway is activated in many viral infections, including Flavivirus, Coxsackie virus B3, Adenovirus and Hepatitis B virus infections [52,53,54,55,56].

Lipid rafts are well known to play an important role in the recruitment and concentration of signaling proteins, involved in viral entry [35] including the PI3K/Akt pathway [57] which is classically compartmentalized within these plasma-membrane microdomains. Indeed, disruption of lipid rafts by MBCD reduces Herpes Simplex virus-type 1 and Japanese Encephalitis virus infection and impairs PI3K/Akt signaling [58,59].

MPXV shares 90% overall sequence homology with VACV. Therefore, it is tempting to speculate that they could have the same mechanisms in viral entry and life cycle [60]. To date, only a few studies have addressed MPXV infection in humans, and no specific cellular receptors for its entry have been identified.

A bioinformatic study has suggested that MPXV could bind to cellular receptors in a way similar to VACV MV and HIV, using PI3K/Akt and GTPase signaling pathways to promote its internalization into host cells [61].

MPXV E8L, A15L and A18L proteins are VACV D8L, A14L and A17L orthologs, respectively, which share the same functions as those in VACV [60]. A recent study has shown using a molecular modelling approach that MPXV E8L protein has a ganglioside-binding motif that could allow interaction with ganglioside GM1, a key component of lipid rafts [62,63]. Gangliosides, which contain sialic acid and are electronegatively charged plasma-membrane components that play an important role in viral infections, including Rotavirus, Simian Virus 40, Paramyxoviruses and Influenza virus [64,65,66,67]. Particularly, the E8L protein, through its electropositive surface, could bind to a cluster of gangliosides GM1 into the lipid raft platform, leading to a modulation of the electrostatic surface potential, which is a key parameter in virus transmission and host interaction [63].

The involvement of lipid rafts in MPXV infection has not yet been explored. However, MPXV could utilize the same receptors involved in VACV entry, eventually localized into lipid rafts, and require their integrity due to their high homology.

## 3. Amphotericin B and Lipid Raft Disruption as Therapeutical Approach for the Treatment of Monkeypox Virus Infection

Different classes of drugs, such as anesthetics, non-steroidal anti-inflammatory, anticancer or antiviral drugs, act on membrane lipid rafts, in addition to their classical direct interaction with membrane receptors, enzymes or ion channels [37]. The therapeutic modulation of lipid rafts has great potential as an antiviral strategy. Lipid-raft-targeting drugs, by altering or disrupting their architecture, can affect several cellular processes organized in the raft platform, such as signal transduction, trafficking or phagocytosis. One of the principal raft components targeted by these drugs is cholesterol, the dynamic glue that maintains raft architecture, and its removal leads to the dissociation of most proteins from rafts, rendering them non-functional. A classical cholesterol-depleting reagent is MBCD, a cyclic heptasaccharide that is frequently used to disrupt lipid raft architecture [68]. In HIV [69], Herpes Simplex virus and Rotavirus infections [70,71], MBCD has been shown to inhibit the viral entry step into host cells, whereas in Japanese encephalitis and Dengue virus infection, it decreased not only viral entry but also an intracellular replication step [72]. MBCD is frequently used as an excipient to facilitate the solubility, bioavailability and stability of many drugs [73], but further studies on the pharmacokinetics and safety of MBCD are needed to propose it as an antimicrobial drug in humans. Filipin, a polyene antibiotic, also inhibits bacterial and viral entry into the cells by depleting cholesterol from lipid rafts. It has a critical limitation due to its cellular damage and is traditionally used as sterol marker [35].

Statins, clinically approved and used for reducing cholesterol levels to prevent cardiovascular diseases, have also been reported to inhibit infections by several Flaviviruses, such as Hepatitis C virus, Dengue virus, West Nile virus and Zika virus [74,75,76,77], although a direct effect on viral biogenesis rather than on host cells has been reported.

Amphotericin B (AmphB) is a macrolide, polyene antibiotic produced from a strain of *Streptomyces nodosus* with a broad range of activity against yeasts and molds, as well as the protozoan parasite *Leishmania* spp. and a low tendency of drug resistance [78]. Resistance to AmB is still rare compared to other antifungal drugs, likely because AmphB targets a major cell membrane component, unlike other antifungals which target an enzyme. AmphB acts via selective binding to ergosterol, a key component of the fungal cell membrane. Eight AmphB molecules bind to eight ergosterol molecules via the polyene hydrophobic chain, leading to the formation of pores on the cell membrane. Pore formation results in K^+^ efflux, fungal glycolysis inhibition, and Mg^++^ efflux with simultaneous proton influx and subsequent cell death. This antifungal has other toxic mechanisms than pore formation at the membrane, such as the induction of oxidative stress in the cells. Importantly, AmphB is also able to bind and sequester mammalian membrane cholesterol, causing damage to the host cells [79]. AmphB is derived from a natural compound with a complex structure and for this reason it has effects other than growth inhibition or the killing of fungi. Interestingly, it has been demonstrated that it has immunostimulatory/immunomodulatory properties due to its binding to toll-like receptors (TLR) 2 and 4 and to CD14 of immune host cells. TLR2 and TLR4 engagement leads to the production of pro-inflammatory and anti-inflammatory cytokines, respectively, whereas CD14 binding by the drug activates the signaling of both TLRs [80,81]. Moreover, this antifungal upregulates the expression of genes involved in angiogenesis [82] and induces the accumulation of nitric oxide [83] and reactive oxygen intermediates [84]. The AmphB effect on the host immune response has been presented as an additional mechanism of action for this antifungal drug, but it has also been related to its known toxicity [79].

Renal toxicity, liver damage and acute infusion-related adverse effects, such as fever and nausea, are associated with intravenous AmphB administration [85]. Despite its toxicity, AmphB remains the first-line treatment for severe and life-threatening systemic fungal diseases and the treatment for visceral leishmaniasis when the parasite develops resistance to antimonial drugs.

We previously demonstrated that AmphB drastically inhibits *C. albicans* phagocytosis by human monocytes through its ability to sequester host-cell membrane cholesterol and disrupt lipid raft architecture [38]. We then expanded our studies to investigate whether AmphB would also block host-cell infection by bacteria and viruses that exploit lipid rafts as an entry platform. We demonstrated that AmphB inhibits *M. tuberculosis* infection of human alveolar epithelial cells [39] and Zika virus infection of Vero cells [40]. Unfortunately, the AmphB deoxycholate formulation is known to have high dose-limiting toxicity, but now, less toxic formulations are available, consisting of liposomes containing hydrogenated soy phosphatidylcholine and cholesterol with AmphB intercalated within the membrane (LipAmphB) [86]. LipAmphB Ambisome^®^ is approved by the FDA for the treatment of several clinical disorders, such as febrile neutropenia, or infections, such as systemic aspergillosis, candidiasis and visceral leishmaniasis [87,88]. LipAmphB can be nebulized without disrupting liposomes, thus allowing AmphB to directly reach the lung [86]. Topical formulations of both AmphB and LipAmphB have been also created for the treatment of skin fungal infections or cutaneous leishmaniasis [89,90]. However, the therapeutic effects of these formulations are a matter of discussion considering both the type of infection and the clinical study. Liposomes are generally considered an optimal delivery system for the skin due to their ability to cross the stratum corneum, reach the dermis, provide prolonged release, protect the drug and allow high local concentrations at the infected site. Delivery of active agents to the skin by liposomes has improved topical therapy in the field of dermatology. Interest in these carriers arises from their ability to include different biological materials and to deliver them to several cell types.

Mpox is primarily transmitted through body fluids and close skin-to-skin or mucosal contact from active lesions; thus, it is tempting to speculate that a topical formulation of AmphB or of LipAmphB would be not only beneficial for the treatment of the lesions, but also particularly appropriate to limit virus spreading and transmission.

## 4. Conclusions

Lipid rafts are the focus of intense research in the field of infection as highlighted by the increased use of lipid-raft-disrupting drugs as antimicrobial agents. Each year, the number of reports implicating new microbes and their interactions with lipid rafts rapidly increases and, in parallel, the development of new drugs targeting host lipid rafts has been extensively studied. Targeting lipid raft membrane domains could represent a new method of drug design and development. The antifungal drug AmphB is able to disrupt lipid raft architecture by its capacity to sequester host-cell membrane cholesterol [38,39,40]; consequently, it could affect Orthopoxvirus infection, which has been shown to be mediated almost in part by the lipid raft platform [47]. Starting from the assumption that MPXV could also exploit lipid rafts for host-cell entry, here we hypothesize that AmphB could inhibit MPXV infection and PI3K/Akt signaling (Figure 1). We thus speculate that this drug could be repurposed for the treatment of Mpox, which represents the most recent emerging zoonotic disease worldwide. Agents that specifically disrupt lipid rafts and deplete rafts’ cholesterol have been proposed to reduce SARS-CoV-2 infectivity and COVID-19 severity because the SARS-CoV-2 host-cell-receptor angiotensin-converting enzyme 2 (ACE2) is localized into lipid rafts [91,92]. In particular, the statin simvastatin has been shown to displace ACE2 on cell membrane lipid rafts, affecting the course of SARS-CoV-2 infection [93]. The approach of targeting specific host-cell membrane structures or functions, instead of virus specific targets, aims to inhibit viral entry and/or replication. This strategy could help to fight, at least in part, the viral multi-drug resistance threat, although viral mutants able to overcome the activity of these compounds are known. A major pitfall of host-targeted antiviral drugs is related to the toxic side effects on host cells as they can target cellular pathways and structures essential for host survival. For this reason, alternative formulations of AmphB are needed to overcome the known toxicity of this antifungal drug.

The Mpox outbreak was declared a “public health emergency of international concern” by the WHO, after H1N1 (2009), Polio (2014), Ebola (2014), Zika (2016), and recently COVID-19 (2020) [94]. Although a newer vaccine based on a modified attenuated vaccinia virus (Ankara strain) was approved for Mpox prevention in 2019, its accessibility is limited and few studies demonstrating its efficacy are available. Therefore, new antiviral drugs for Mpox are urgently needed and repurposing an old drug could be an interesting approach due to the emergency of the recent situation. There is great interest in drug repurposing to accelerate the discovery of agents that can cure or prevent infectious outbreaks and that have already proven to be safe in humans. With this approach, the risk of failure is highly reduced, and the toxicity aspects associated with these drugs can be overcome to a great extent. Development of novel antiviral molecules from the beginning has tremendous high costs and the translation from bench to bedside takes about 10–15 years. The drug repurposing approach can reduce the duration of the different steps of preclinical evaluations and clinical trials. The potential of lipid rafts as targets for antiviral interventions opens doors to new ideas in drug repurposing.

## Data Availability

Not applicable.
